# Complete Genome Sequence of Enterotoxigenic Escherichia coli Siphophage Seurat

**DOI:** 10.1128/genomeA.00044-15

**Published:** 2015-02-26

**Authors:** Dung P. Doan, Lauren E. Lessor, Adriana C. Hernandez, Gabriel F. Kuty Everett

**Affiliations:** Center for Phage Technology, Texas A&M University, College Station, Texas, USA

## Abstract

Enterotoxigenic Escherichia coli (ETEC) is one of the leading causes of diarrhea in developing countries. Bacteriophage therapy has the potential to aid in the prevention and treatment of ETEC-related illness. To that end, we present here the complete genome of ETEC siphophage Seurat and describe its major features.

## GENOME ANNOUNCEMENT

Enterotoxigenic Escherichia coli (ETEC) is one of the leading causes for infectious gastroenteritis that results in approximately 120,800 annual deaths worldwide (1). The rise in antibiotic resistance has prompted the need for alternative treatments of pathogenic bacterial infections, such as bacteriophage therapy (2, 3). Here, we describe the complete genome of bacteriophage Seurat, a siphophage infecting ETEC. Genome analysis suggests Seurat is a lytic phage and therefore could play a role in phage therapy to reduce the number of deaths due to ETEC infection per year.

Seurat was isolated from a sewage sample collected in College Station, TX. Phage DNA was sequenced using 454 pyrosequencing at the Emory GRA Genome Center (Emory University, GA). Trimmed FLX Titanium reads were assembled to a single contig at 107.3-fold coverage using Newbler assembler, version 2.5.3 (454 Life Sciences) at default settings. The contig was confirmed to be complete by PCR using primers that face the upstream and downstream ends of the phage DNA. Products from the PCR amplification of the junctions of concatemeric molecules were sequenced by Sanger sequencing (Eton Bioscience, San Diego, CA). Genes were predicted using GeneMarkS (4) and corrected using software tools available on the Center for Phage Technology (CPT) Galaxy instance (https://cpt.tamu.edu/galaxy-public/). Transmission electron microscopy was performed at the Texas A&M University Microscopy and Imaging Center.

Seurat has a genome of 56,776 bp with 88 coding sequences, a 44.6% G+C content, and a 93.2% coding density. It shows 10.5% average nucleotide identity to E. coli phage 9g (GenBank accession no. NC_024146), which is not enough to justify clustering as described by Grose and Casjens (5). Seurat can therefore be designated a novel singleton cluster containing a single phage.

DNA synthesis machinery includes genes coding for DNA polymerase, sliding clamp subunit, helicase, and primase. Genes for morphogenesis proteins were identified (capsid, tape measure, tape measure chaperone, tail fibers). DNA packaging genes include *terS* and *terL*. Comparison of the Seurat TerL protein to TerL proteins of known packaging strategies suggests that Seurat uses a pac-headful DNA packaging mechanism. For annotation purposes, the genome has been opened to the small terminase gene (6). Seurat also encodes a dUTPase to ensure low levels of cellular dUTP through the hydrolysis of dUTP into dUMP and PP_i_ (7).

Seurat encodes queuosine biosynthesis proteins, QueC, QueD, QueE, and FolE. Queuosine, a hyper-modified guanine nucleoside, plays an important role in stabilizing the anticodon-codon interaction at the wobble position of certain tRNAs to improve translation accuracy (8). QueCDE and FolE are involved in the biosynthesis of queuosine precursors preQ_0_ and preQ_1_ (9, 10). The most complete queuosine biosynthesis pathway reported in a bacteriophage thus far has been in the Streptococcus pneumoniae phage Dp-1 (NC_015274) (11). Comparison of the queuosine pathways between the two phages shows that Seurat is missing QueT, a transporter, and QueF, a precursor enzyme.

### Nucleotide sequence accession number.

The genome sequence of phage Seurat was contributed as accession no. KM236243 to GenBank.
